# Comparison of computed tomographic imaging-guided hook wire localization and electromagnetic navigation bronchoscope localization in the resection of pulmonary nodules: a retrospective cohort study

**DOI:** 10.1038/s41598-020-78146-z

**Published:** 2020-12-08

**Authors:** Yu Tian, Cong Wang, Weiming Yue, Ming Lu, Hui Tian

**Affiliations:** 1 Department of Thoracic Surgery, The Second Hospital of Shandong University, Jinan, 250000 People’s Republic of China; 2 Department of Radiation Oncology, Qilu Hospital, Shandong University, Jinan, 250012 People’s Republic of China; 3 Department of Thoracic Surgery, Qilu Hospital, Shandong University, 107# Wenhua Xi Road, Jinan, 250012 People’s Republic of China; 4 School of Medicine, Shandong University, Jinan, 250012 People’s Republic of China

**Keywords:** Non-small-cell lung cancer, Cancer therapy, Lung cancer

## Abstract

The resection of nodules by thoracoscopic surgery is difficult because the nodules may be hard to identify. Preoperative localization of pulmonary nodules is widely used in the clinic. In this study, we retrospectively compared CT-guided hook wire localization and electromagnetic navigation bronchoscopy (ENB) localization of small pulmonary nodules before resection. Patients who underwent localization with CT-guided hook wire or ENB followed by video-assisted thoracoscopic surgery (VATS) at Qilu Hospital of Shandong University between January 2016 and December 2019 were retrospectively included. Clinical parameters, complication and failure rate, and localization time were compared between two groups. A total of 157 patients underwent the localization procedure successfully. Pulmonary nodules were localized by CT-guided hook wire in 105 patients and by ENB in 52 patients. The nodule size in ENB group was smaller than that in CT-guided localization group (*P* < 0.001). Both CT-guided localization and ENB localization were well tolerated in all patients, while ENB localization leaded to less complications (*P* = 0.0058). In CT-guided localization group, 6 patients failed to be located while none failed in ENB group (*P* = 0.079). The procedure time was 15.15 ± 3.70 min for CT-guided localization and 21.29 ± 4.00 min for ENB localization (*P* < 0.001). CT-guided localization is simple and feasible for uncertain pulmonary nodules before surgery. ENB localization could identify small lung nodules with high accuracy and achieve lower incidence of complications.

## Introduction

Lung cancer is the most lethal disease worldwide. It accounts for one-quarter of all cancer deaths around the world. Although the diagnosis and treatment technologies have improved rapidly in recent years, the prognosis of lung cancer remains poor. The 5-year survival rate of lung cancer is only 18%, which is the lowest among all kinds of cancers^1^. With widespread use of computed tomography (CT) in the clinic, the discovery of small or faint lesions on CT become fesible^2^. However, the resection of nodules by thoracoscopic surgery is difficult because the nodules may be hard to identify, especially for deep pulmonary small nodules. If the location of nodules cannot be accurately determined during the operation, it will increase the possibility of thoracotomy. Currently, preoperative localization of pulmonary nodules is widely used in the clinic, including CT-guided transthoracic approach, hook-wire and coil embolization. However, these methods increase radiation exposure and cause complications^3^.

Electromagnetic navigation bronchoscope (ENB) could reach further peripheral lung compared to conventional electronic bronchoscopy^4,5^. Combined with the path constructed by ENB, we can mark the location of small pulmonary nodules. This method has better safety and effectiveness. Several studies have reported that ENB-guided transbronchial needle biopsy achieves better accuracy and lower complication rates, compared to conventional percutaneous core needle biopsy^6–8^. In this study, we retrospectively compared CT- and ENB-guided localization of small pulmonary nodules before resection.

## Methods

We retrospectively analyzed patients who underwent localization with CT or ENB followed by Video-assisted thoracoscopic surgery (VATS) at Qilu Hospital of Shandong University from January 2016 to December 2019. Patients were included based on the following criteria: patients with only one pulmonary nodule smaller than 10 mm, preoperative localization were conducted, undergone VATS after localization and received pathology diagnosis. Clinical parameters were recorded, including the gender, age, smoking status, histology, and stage. CT findings were recorded, including lesion size, location, density, and distance from pleural distance (PD). The nodule size was measured directly on CT images, and the longest diameter was the nodule size. The nodule was classified according to the density as pure nodular ground-glass opacity (GGO), 0–50% GGO and > 50% GGO.

### Percutaneous CT-guided localization

**Figure 1:**
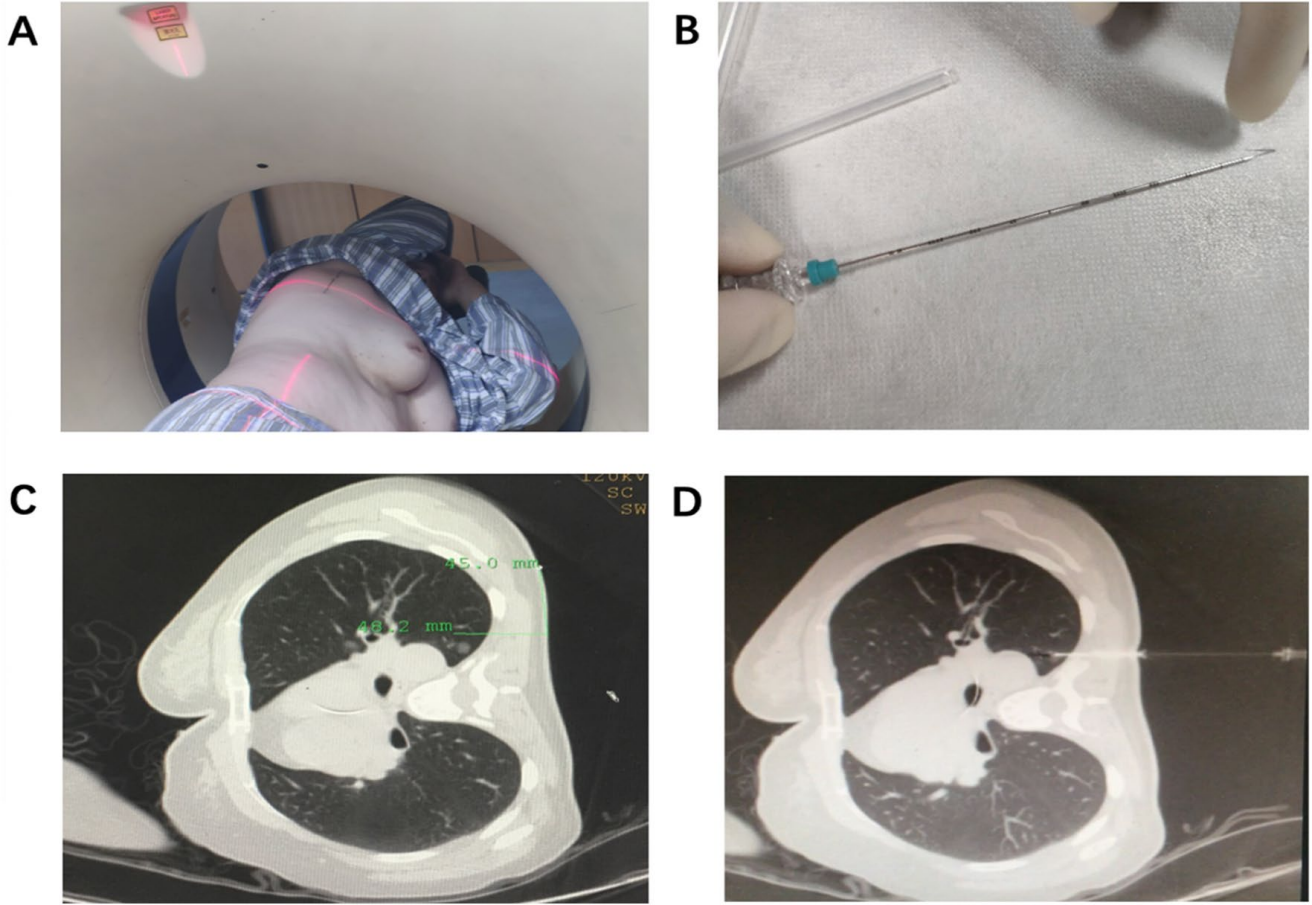
(**A**) Coronal localization by CT and Sagittal marking with steel needles. (**B**) The picture of steel needle. (**C**) Measuring the distance of puncture point and locating the distance between steel needle and puncture point in coronal position of CT image. (**D**) The results of puncture were confirmed by CT and the complications were detected.

CT scan was performed to confirm the presence of nodules before the localization. We used metal markers on the body surface to determine the missing line. Then we used CT and the laser to determine the coronal line. Coronal line represents the CT coronal plane of the lesion, and the intersection of two lines is used to determine the specific location of the nodule (Fig. 1).

### ENB planning and surgical procedures

**Figure 2:**
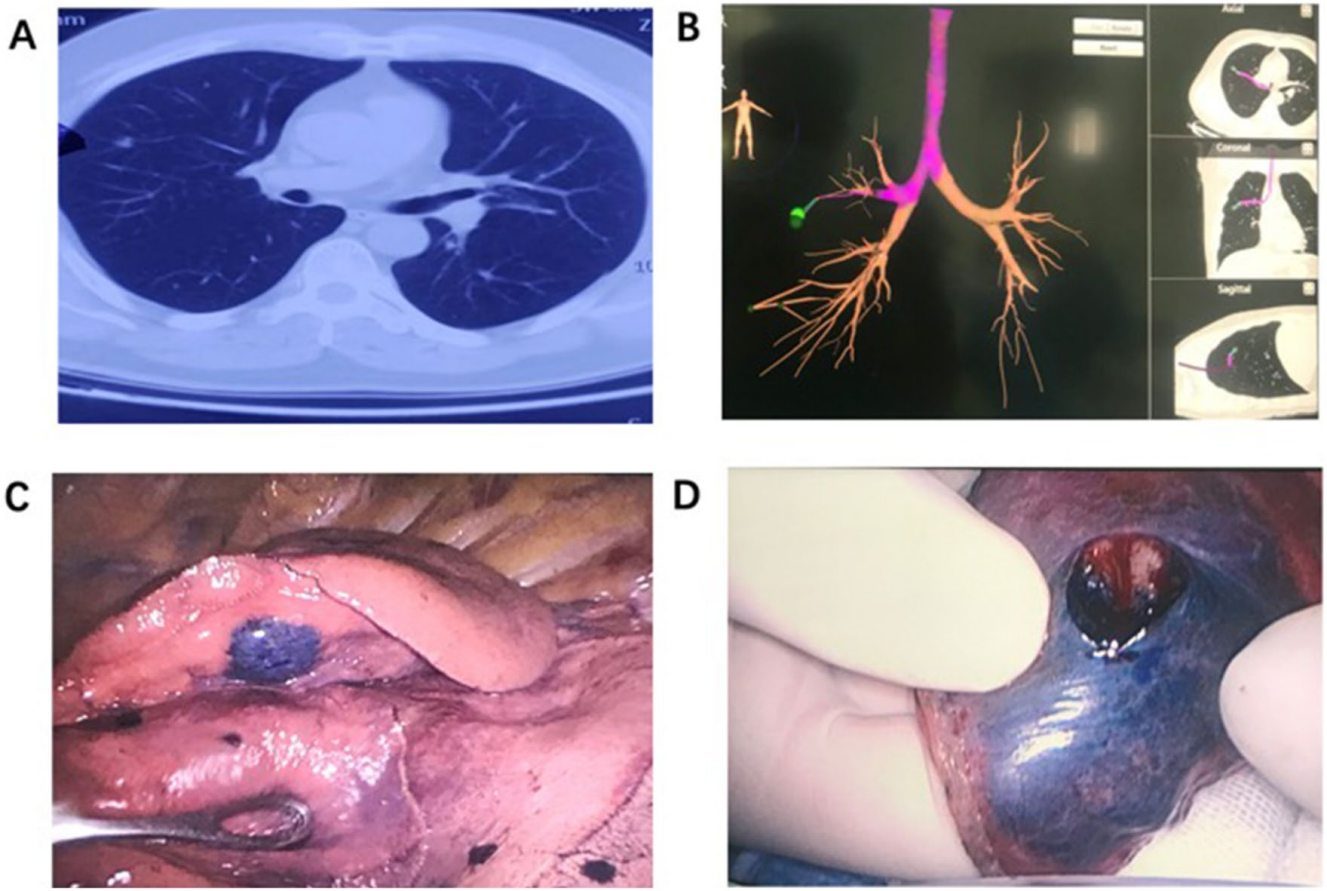
(**A**) A small pulmonary nodule is located in the right upper lobe. (**B**) Before operation, magnetic navigation software was used to design magnetic navigation localization path. (**C**) According to the designed path, the magnetic navigation probe was stained with methylene blue after reaching the lesion location. (**D**) The accuracy of localization was verified after thoracoscopic surgery.

ENB-guided dye-marking was performed for subsolid nodules less than 10 mm near the pleura, or nodules with volume less than 20 mm located more than 10 mm from the pleural surface. The axial, sagittal and coronal views of CT images were used for planning using super Dimension system (Medtronic, Minneapolis, MN, USA). After general anesthesia with intubation, patients were navigated using the 7th edition Super Dimension Navigation System (Covidien, Minneapolis, MN, USA) to localize and plan a route to the nodules one day before the operation. (Fig. 2).

Thoracoscopic pulmonary surgery was performed immediately after the completion of magnetic navigation surgery. The primary lung cancer and lymph nodes were dissected by VATS. All patients underwent segmental or combined segmental resection, and the pathological examination was performed quickly after operation. All patients received postoperative care. The clinicopathological data, procedure parameters, and complications were evaluated.

This study was approved by the Ethics Committee of Qilu Hospital of Shandong University. The research was performed in accordance with relevant guidelines. Informed consent was obtained from all participants.

### Statistical analyses

Measurement data were presented as mean ± standard deviation and analyzed by ANOVA method. Count data were presented as percentage and analyzed by Chi-square test. Statistical analysis was performed using SPSS 23.0 software (SPSS Inc., Chicago, Il, USA). *P* < 0.05 was regarded as significance.

## Results

### Characteristics of the patients

**Figure 3:**
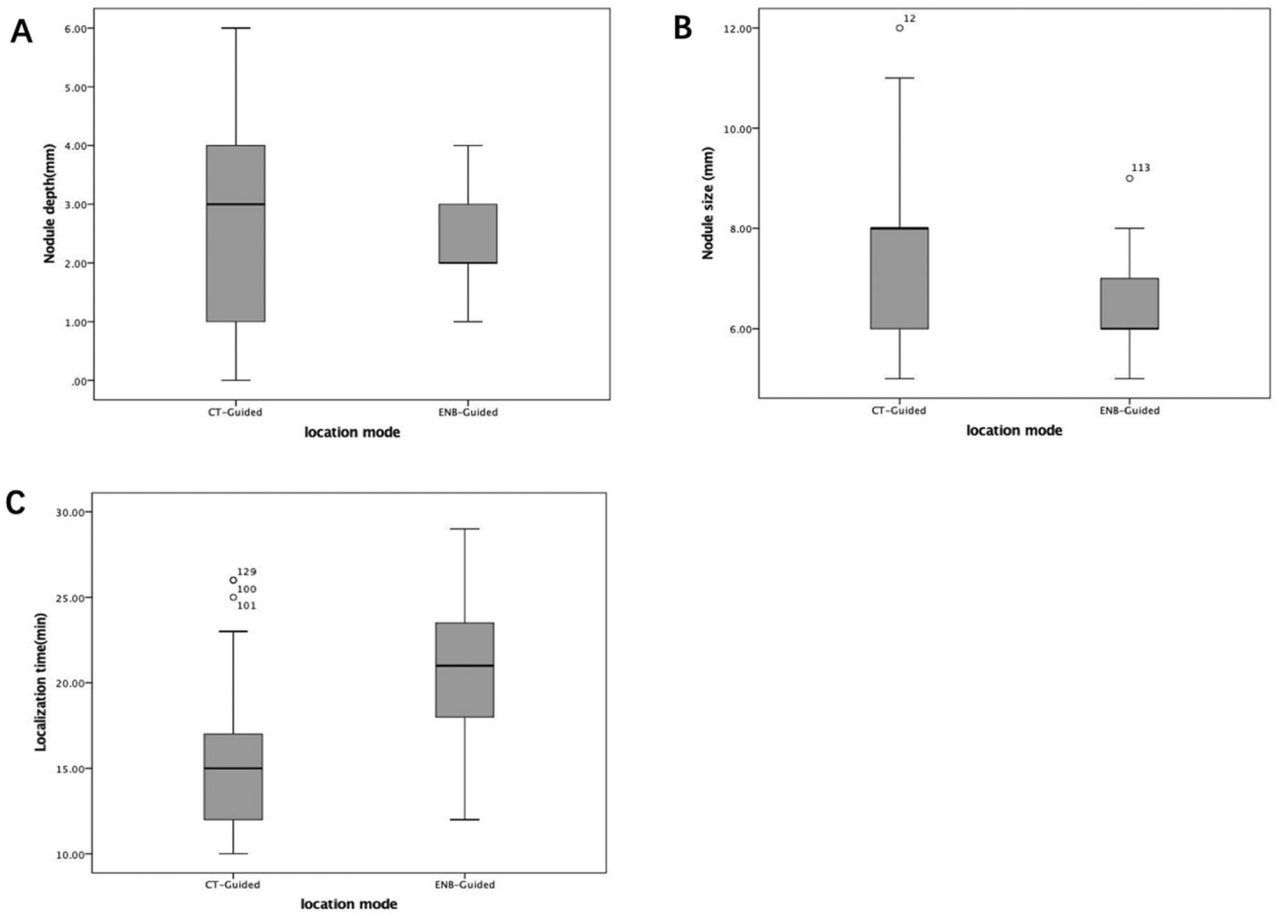
Characteristics of pulmonary nodular lesion in two localization groups. (**A**) Nodule depth. (**B**) Nodule size. (**C**) Localization time.

We included a total of 157 patients (88 males and 69 females) who underwent the localization procedure successfully. The nodules were localized by CT guidance in 105 patients and by ENB in 52 patients. Among 157 patients, 95 were smokers and 62 were non-smokers. Clinical characteristics of the patients and lung nodules were listed in Table 1. The mean value of nodule size was 6.99 ± 1.37 mm (range 5.0–12.0 mm). The nodule size of ENB localization was smaller than that of CT-guided localization (*P* < 0.001). The nodule depth in ENB localization group was smaller than CT-guided localization (*P* = 0.007). Each patient had only one nodule, and the 157 nodules included 74 pure GGO nodules, 66 part solid nodules with GGO < 50%, and 17 part-solid nodules with GGO > 50%. The nodules distributed in each lobe of the lung. Sixteen nodules were in the left upper lobe, 26 nodules were in the left lower lobe, 58 nodules were in the right upper lobe, 17 nodules were in the right middle lobe, and 40 nodules were in the right lower lobe. The pathology diagnoses of 157 nodules were as follows: 29 benign nodules, 76 adenocarcinomas in situ (AIS), 44 minimally invasive adenocarcinomas (MIA) and 8 invasive adenocarcinomas (IA). Standard lobectomy was performed again in 8 IA patients. pTNM stages of 128 malignant tumors were T1aN0M0 (Fig. 3).

**Table 1:** Clinical characteristics and demographics of two localization groups.

Variables	CT-guided hook wire localization	ENB localization	P*
Age			0.459
≥ 60 years old	51	22	
< 60 years old	54	30	
Gender			0.527
Male	57	31	
Female	48	21	
Smoking			0.116
No	46	16	
Yes	59	36	
Location			0.066
RUL	44	14	
RML	11	6	
RLL	20	20	
LUL	13	3	
LLL	17	9	
Nodule size			< 0.001
< 6 mm	8	11	
6–8 mm	43	38	
8–10 mm	54	3	
Nodule depth			0.007
0 cm	3	0	
0–3 cm	61	43	
> 3 cm	41	9	
Nodule density			0.567
Pure GGO	47	27	
> 50% GGO	45	21	
< 50% GGO	13	4	
Pathological diagnosis			0.117
Benign	15	14	
AIS	50	26	
MIA	33	11	
IA	7	1	

*Chi-square test.

### Localization of the nodules

Both CT-guided and ENB localization were well tolerated in all patients, without mortality or major complications, such as tension pneumothorax, massive hemoptysis and active intrathoracic hemorrhage. In CT-guided localization group, the incidence of asymptomatic hemopneumothorax was 7.6% (8/105), symptomatic hemopneumothorax was 3.8% (4/105), hemoptysis was 0.9% (1/105) and decoupling was 0.9% (1/105). ENB localization leaded to less complications (0/52) compared with CT-guided localization (14/105) (*P* = 0.0058). In addition, in CT-guided localization group, 6 patients failed to be located due to deviation of the puncture hook position, while none failed in ENB group (*P* = 0.079). The procedure time for CT-guided localization was 15.15 ± 3.70 min (range 10–26 min), while the procedure time for ENB localization was 21.29 ± 4.00 min (range 12–29 min) (*P* < 0.001). In CT-guided hook wire localization group, 6 hook wires passed through the nodule and 99 hook wires passed beside the nodule. The time of needle-carrying time for patients receiving CT-guided localization was 37.12 ± 17.29 min (*P* < 0.001) (Table 2, Fig. 3).

**Table 2:** Localization and surgery results in the ENB and CT groups.

Variables	CT-Guided Localization	ENB	P*
Complications			0.0058
No complications	91	52	
With complications	14	0	
Asymptomatic hemopneumothorax	8	0	
Symptomatic hemopneumothorax	4	0	
Hemoptysis	1	0	
Decoupling	1	0	
Failed to localization	6	0	0.079
Localization time (min)	15.15 ± 3.70	21.29 ± 4.00	< 0.001
Hook wire station			–
Through the nodule	6	–	
Beside the nodule	99	–	
Needle-carrying time (min)	37.12 ± 17.29	0	< 0.001

*Chi-square test.

## Discussion

The application of high resolution CT increases the rate of detecting small pulmonary lesions. Therefore, it is important to accurately locate the sublobar or non-anatomical resection using a variety of approaches^9–12^. Currently used localization methods have several advantages and disadvantages. For example, percutaneous hook wire implantation has the advantages of simple operation, accurate localization and less complications, however, it is an invasive operation. ENB, a new technique developed recently, could accurately access peripheral lung lesions beyond the reach of conventional bronchoscopy. ENB-guided localization could reduce complications such as pneumothorax, hemothorax and hemoptysis, compared with traditional localization methods. Therefore, ENB-guided localization is regarded as a promising tool in thoracic surgery^13,14^. In this study, we compared CT-guided localization and ENB localization for lung nodules in patients.

In our study, 52 patients in ENB localization group had no complications, while 14 in 105 patients with CT-guided puncture hook localization had complications. The most common complication was hemopneumothorax and no catastrophic complications occurred. ENB localization technology achieved higher safety and operability, because all operations can be carried out in the operating room. Patients in CT-guided puncture hook localization group needed to be transported from CT room to operating room, which increases the risk of whole localization process. In addition, 6 (5.7%) patients in CT-guided localization group failed to be located, mostly because of the deviation of the puncture hook position. Localization time was longer in ENB group because path setting has to be conducted before localization. ENB achieves lower complication and failure rate and increases localization time compared with CT-guided puncture hook localization.

As for the size of pulmonary nodules in our inclusion criteria, we only included nodules less than 10 mm. For 6–12 mm pulmonary nodules, the volume or solid components increased during the follow-up period according to NCCN guidelines. Patients with pulmonary nodules ≥ 12 mm should be diagnosed and treated as early as possible according to the Chinese Consensus on diagnosis and treatment of pulmonary nodules. If the diagnosis cannot be confirmed, multidisciplinary team (MDT) discussion is recommended. It is difficult to locate the pulmonary nodules smaller than 10 mm in clinical operation. Therefore, the size of pulmonary nodules in our retrospective study is smaller than 10 mm.

The operation cannot be performed after localization immediately in CT-guided hook wire localization method. Patients need to wait for surgical resection with a puncture hook which increases the complexity and risks of the operation. However, the procedure does not require additional facilities such as radiotracer, contrast injection or coil insertion. Therefore, CT-guided puncture hook localization is widely used in clinical applications for this method is simple, feasible and less costly. ENB method requires more labor and experience to master and is low cost-effectiveness, while it achieves smaller trauma, more accurate localization and higher safety. ENB also has potential to conduct interventional lung surgery^13,15–18^. Therefore, ENB localization technology has a good application prospect.

## Conclusions

In conclusion, CT-guided localization is simple and feasible for uncertain pulmonary nodules before surgery. ENB localization could identify small lung nodules with high accuracy and low incidence of complications.

## Abbreviations

CT: Computed tomographic imaging
ENB: Electromagnetic navigation bronchoscopy
VATS: Video-assisted thoracoscopic surgery
PD: Distance from pleural distance
GGO: Ground-glass opacity
AIS: Adenocarcinoma in situ
MIA: Minimally invasive adenocarcinoma
IA: Invasive adenocarcinoma
